# What if it was easier to prevent schizophrenia than to treat it?

**DOI:** 10.1038/s41537-017-0012-x

**Published:** 2017-02-13

**Authors:** Kristen J. Brennand

**Affiliations:** 1 Departments of Psychiatry and Neuroscience, Icahn School of Medicine at Mount Sinai, 1425 Madison Avenue, New York, NY 10029 USA; 2 Friedman Brain Institute, Icahn School of Medicine at Mount Sinai, 1425 Madison Avenue, New York, NY 10029 USA

Neural tube defects occur when the brain or the spinal cord fails to close early in embryonic development. While genetic polymorphisms affecting folate metabolism suggest that only certain individuals may be at increased risk for neural tube defects,^1^ since 1992 all women of childbearing age have been recommended to consume daily folic acid.^2^ This proved so effective that wheat products are now fortified as well.^2^ Our ability to prevent neural tube defects, with only a vitamin, demonstrates that we must now consider the idea that we can preclude other neurodevelopmental disorders as well.

Schizophrenia is preceded by a long period of disease progression prior to the onset of symptoms. How best to study a disease prior to diagnosis? Classically, physicians and scientists have done this by tracking high-risk individuals for decades, waiting for symptom onset to occur in a small subset of their cohort. This is both time-consuming and inefficient. With the Nobel Prize winning discovery by Shinya Yamanaka in 2007, it is now possible to reprogram human-induced pluripotent stem cells (hiPSCs) from patient cells;^3^ these hiPSCs have the ability to differentiate into all cell types found in the body.^4^ Suddenly, scientists gained the ability to generate stem cells from every person on the planet, providing the opportunity to study disease processes in patient-derived cells cultured in a laboratory dish.

Current hiPSC differentiation strategies yield neurons that mostly resemble the fetal brain cells (Brennand *et al.* 2015 Molecular Psychiatry; Mariani *et al.* 2012 PNAS; Nicholas *et al.* 2013, Cell Stem Cell; Pasca *et al.*, Nature Methods 2015), making them a better tool for the study of the molecular aspects of disease predisposition, rather than the disease-state itself. For example, hiPSC-based studies of late onset neurodegenerative diseases such as Parkinson’s Disease,^5–8^ Alzheimer’s Disease^9, 10^ and amyotrophic lateral sclerosis^11^ have failed to recapitulate the severe neuronal loss observed in human disease. Using hiPSCs, we and others have found that schizophrenia hiPSC-derived neural progenitor cells show evidence of aberrant migration,^12^ deficits associated with adherens junctions and polarity,^13^ increased oxidative stress^12, 14, 15^ and perturbed responses to environmental stressors;^16^ while schizophrenia hiPSC-derived neurons exhibit decreased neurite number,^17^ reduced synaptic maturation^14, 17–19^ and synaptic activity,^18, 19^ and blunted activity-dependent response.^20^ These in vitro deficits may reflect processes underlying disease predisposition in patients. Consistent with this, we recently reported unbiased hiPSC-based evidence^21^ that was convergent with novel human genetics-based analyses,^22^ suggesting that microRNA-9 may partially contribute to genetic risk for schizophrenia in a subset of patients.

While the potential of hiPSC-based models to establish a personalized medicine approach to the treatment of schizophrenia—one drug screen per genotype—has been widely discussed,^23^ here I posit that the first hiPSC-based screens may instead identify drugs more suitable for disease prevention. It may ultimately prove easier to apply hiPSC-based models towards the prevention, rather than treatment, of schizophrenia.
